# Interfacial Salt Redistribution Enables Rapid Atmospheric Water Harvesting in a Biomimetic Multi‐Layer Hygroscopic Membrane

**DOI:** 10.1002/advs.78196

**Published:** 2026-10-06

**Authors:** Fu‐Yao Zhong, Chao‐Yang Guo, Zai‐Dong Shao, Lu‐Bing Zhong, George Zheng Chen, Yaoyao Zheng, John L. Zhou, Yu‐Ming Zheng, Jun He

**Affiliations:** ^1^ State Key Laboratory of Advanced Environmental Technology Institute of Urban Environment Chinese Academy of Sciences Xiamen Fujian China; ^2^ Department of Chemical and Environmental Engineering Climate and Environmental Solution Research Center University of Nottingham Ningbo China Ningbo Zhejiang China; ^3^ Department of Chemical and Environmental Engineering University of Nottingham Nottingham UK; ^4^ Faculty of Engineering University of Nottingham Nottingham UK; ^5^ Faculty of Science and Engineering University of Nottingham Ningbo China Ningbo China

**Keywords:** atmospheric water harvesting, biomimetic hygroscopic membranes, brush‐like hydrogels, interfacial deliquescence, mass transport engineering, sorption‐based water capture

## Abstract

Sorption‐based atmospheric water harvesting (SAWH) offers a promising route for decentralized freshwater production, but practical water output is often limited by slow sorption kinetics in salt‐loaded hydrogel sorbents. Here, a biomimetic multi‐layer heterogeneous hygroscopic (MHH) membrane is developed to decouple vapor capture from bulk liquid storage. Inspired by the salt‐management strategy of *Avicennia marina* leaves, the membrane integrates an air‐accessible nanofiber interface with a brush‐like hydrogel reservoir. During drying, lithium chloride (LiCl) becomes enriched within the nanofiber layer, enabling rapid interfacial deliquescence upon exposure to humid air. The resulting salt solution is then transferred into the underlying hydrogel, where polyethylene glycol (PEG) side chains promote water transport by reducing internal diffusion resistance. The MHH membrane reaches approximately 85% of its equilibrium uptake within 90 min at 70% relative humidity (RH), while maintaining a water uptake of approximately 2.4 g g^−1^. In a semi‐continuous three‐bed SAWH device, the membrane delivers 3.47 L m^−2^ over 8 h at 25°C and 90% RH and demonstrates outdoor operation under naturally variable conditions. This work establishes interfacial salt redistribution coupled with polymer‐network engineering as an effective strategy for fast‐cycling atmospheric water harvesting.

## Graphical abstract

A biomimetic multi‐layer hygroscopic membrane redistributes salt toward an air‐accessible nanofiber interface, enabling rapid interfacial deliquescence while a brush‐like hydrogel promotes efficient liquid transport and storage. By decoupling vapor capture from bulk water storage, the membrane achieves fast sorption kinetics and enables rapid‐cycling, semi‐continuous atmospheric water harvesting.

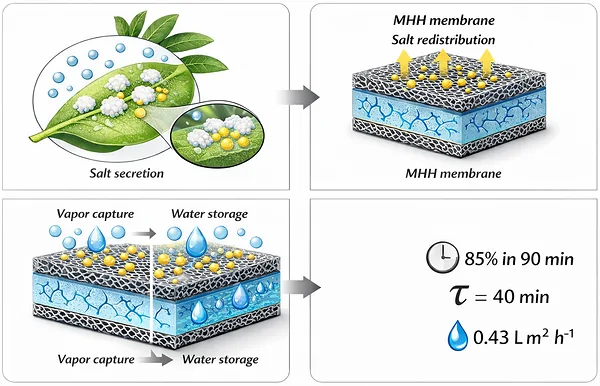

## 1 Introduction

Water scarcity is increasingly intensified by population growth, industrial expansion, and climate change, which together place sustained pressure on accessible freshwater resources [1, 2]. Conventional water‐supply technologies, including desalination and groundwater extraction, can provide large water outputs but are often constrained by high energy demand, geographical dependence, infrastructure requirements, or environmental impacts. Atmospheric water harvesting (AWH), which extracts water directly from ambient air, has therefore attracted growing interest as a decentralized route for freshwater production [3]. Among the available AWH strategies, condensation‐based systems and sorption‐based systems have both been widely investigated. Although recent advances have improved the efficiency of condensation‐based water collection [4, 5], these systems generally require active cooling and considerable electrical input because water vapor condensation involves a large latent heat. Sorption‐based atmospheric water harvesting (SAWH) is attractive because sorbent regeneration can be driven by solar energy or other low‐grade heat sources, making it potentially suitable for off‐grid and resource‐limited settings [6, 7, 8, 9, 10, 11].

Beyond conventional decentralized freshwater production, hygroscopic atmospheric water harvesting is increasingly being explored for application‐specific water supply in agriculture, portable systems, and remote or marine environments. In smart agriculture, sorption‐based water harvesting can be coupled with solar‐powered autonomous irrigation, enabling atmospheric moisture to be captured during favorable periods and subsequently released for plant cultivation. A representative autonomous SmartFarm system has demonstrated this concept for rooftop agriculture, illustrating the potential of atmospheric water harvesting to reduce dependence on conventional irrigation‐water supplies [12]. Recent natural‐fiber‐supported hygroscopic sorbents further demonstrate opportunities for lightweight, scalable, and textile‐integrated atmospheric water‐harvesting systems, broadening the potential for portable and distributed deployment [13]. Hygroscopic materials are also attractive for coastal, island, and marine environments, where the air above seawater can provide an abundant moisture source while access to freshwater infrastructure may be limited. Solar‐triggered harvesting of moisture‐rich air directly above the sea surface has been demonstrated using a super‐hygroscopic hydrogel, highlighting a potential route for freshwater generation in marine and coastal settings [14]. More broadly, recent advances in hygroscopic salt‐embedded composite materials emphasize that practical SAWH development should consider not only sorption capacity but also cycling kinetics, environmental adaptability, scalable device integration, and application‐specific operating requirements [15].

For SAWH materials, water production is governed not only by equilibrium water uptake but also by the rate at which that uptake can be reached [14]. A high‐capacity sorbent is not necessarily productive if it requires a long adsorption time, because the cycle frequency and the daily water yield are then limited. Hygroscopic salt‐composite hydrogels have been extensively explored because they combine the strong water affinity of salts with the water‐storage capacity and structural tunability of polymer networks [15, 16, 17, 18, 19]. However, increasing the salt content to raise water uptake commonly aggravates mass‐transfer resistance within the hydrogel matrix. As a result, salt‐composite hydrogels often face a practical compromise between large water‐storage capacity and fast sorption kinetics.

This kinetic limitation originates from coupled vapor and liquid transport processes [20]. Water vapor must first reach hygroscopic sites, while the deliquesced salt solution must then redistribute through the hydrogel network. Previous analyses have shown that, under representative conditions, hydrogel sorption can be more sensitive to diffusion within the polymer network than to external vapor transport, indicating that internal liquid transport is often a dominant resistance [21]. Considerable effort has therefore been devoted to porous hydrogel architectures that shorten diffusion distances and increase accessible transport pathways [22, 23, 24, 25]. Ice‐templating has been used to prepare porous polyacrylamide hydrogels with improved sorption kinetics [26, 27], foaming has been used to introduce macroporous structures [28], and directional freeze‐drying has been adopted to construct aligned anisotropic channels [29].

Despite these advances, porous‐structure engineering does not fully resolve the salt‐accessibility problem. Freeze‐drying, cryogenic processing, and related templating approaches can be energy‐intensive and time‐consuming [30, 31, 32, 33], and foaming procedures may add processing complexity [34]. More importantly, pore tortuosity and structural heterogeneity can still impose non‐negligible diffusion pathways even in optimized porous networks [27, 35]. In many reported salt‐hydrogel composites, a substantial fraction of the hygroscopic salt remains embedded in the polymer matrix, where direct contact with ambient vapor is limited. A material architecture that relocates hygroscopic salt to an air‐accessible interface while retaining a separate hydrogel domain for liquid storage could therefore address the capacity‐kinetics trade‐off more directly.

A useful biological clue is provided by the mangrove Avicennia marina, which survives in saline environments through controlled salt secretion and water management [36, 37]. Salt ions are transported from internal tissues to specialized glands on the leaf surface, where they accumulate in microstructured regions (Figure 1a,b,d). Under humid conditions, the surface salt can deliquesce and absorb moisture from the atmosphere (Figure 1c). For the present materials design, the most relevant feature is not a literal replication of plant physiology but the spatial separation between internal salt transport/storage and surface‐exposed hygroscopic sites. This separation allows salt to interact directly with ambient vapor at the leaf surface, suggesting a strategy for accelerating moisture capture in artificial sorbents.

**FIGURE 1:**
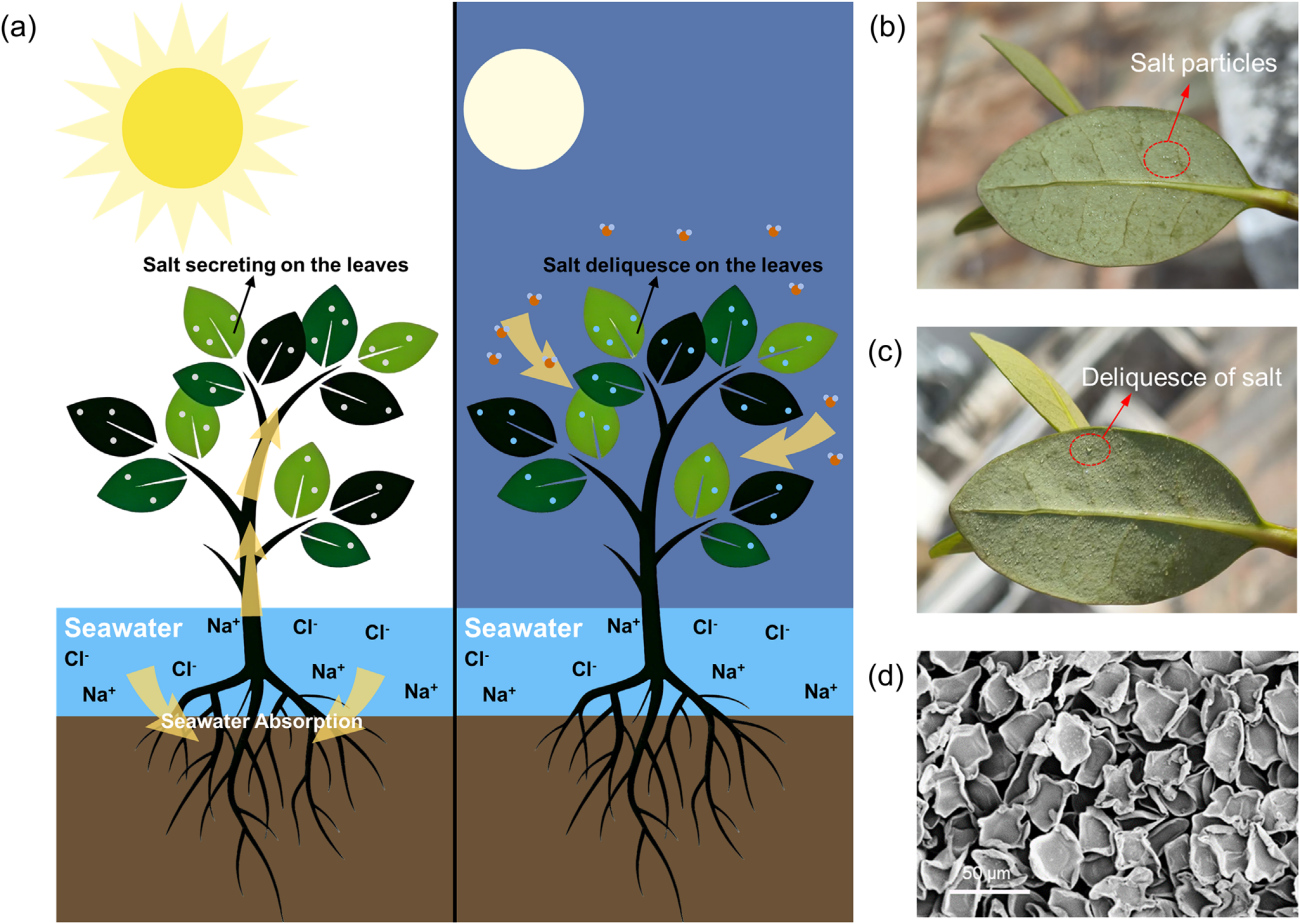
Salt‐secreting behavior of Avicennia marina. (a) Schematic diagram of Salt‐secreting and moisture absorption mechanisms in Avicennia marina. (b) Avicennia marina leaves with salt particles. (c) Avicennia marina leaves with deliquescence salt solution droplets. (d) Microstructure of salt glands on Avicennia marina leaves.

Inspired by this principle, we designed a multi‐layer heterogeneous hygroscopic (MHH) membrane in which vapor capture and bulk liquid storage are spatially separated. The membrane consists of alternating nanofiber and hydrogel layers. The nanofiber layer provides an air‐accessible interfacial region that can accommodate salt crystallization after drying, whereas the hydrogel layer serves as the main reservoir for the absorbed liquid water. During desorption, salt solution within the hydrogel is driven toward the nanofiber domain and crystallizes in the inter‐fiber voids. During the subsequent adsorption step, the surface‐enriched salt can deliquesce rapidly through direct contact with humid air, and the generated salt solution is absorbed into the underlying hydrogel. In this way, architecture decouples initial vapor‐salt interaction from longer‐range liquid storage and reduces the dependence of early‐stage sorption on diffusion through the bulk hydrogel.

Efficient transfer of the deliquesced salt solution into the hydrogel remains essential once interfacial deliquescence begins. To reduce this internal transport resistance, we further introduce a brush‐like hydrogel network containing polyethylene glycol (PEG) side chains. The flexible PEG segments are expected to increase chain mobility and accessible free volume within the polymer network, thereby facilitating water transport and reducing liquid diffusion resistance [38]. The resulting design combines interfacial salt redistribution with molecular‐level hydrogel engineering, providing a coordinated route to accelerate both vapor capture and liquid uptake.

Here, we report a biomimetic MHH membrane that integrates an air‐accessible salt‐enriched nanofiber interface with a brush‐like hydrogel reservoir. The structural design enables rapid moisture absorption while maintaining high water uptake capacity and scalable fabrication through electrospinning and spraying. The following results establish how the layered architecture, salt distribution, polymer‐network mobility, and device‐level cycling collectively determine SAWH performance.

## 2 Experimental Section

### 2.1 Materials

Polyacrylonitrile (PAN), poly(ethylene glycol) acrylate/methacrylate (PEGMA, Mn = 600 Da), acrylamide (AM), N,N‐dimethylformamide (DMF), potassium persulfate (KPS), poly(ethylene glycol) diacrylate (PEGDA, Mn = 200 Da), cyclohexane, Span 80, lithium chloride (LiCl), pyrrole, ammonium persulfate (APS), hydrochloric acid (HCl), photoinitiator 2959, and basic alumina were purchased from Aladdin, Macklin, or Sinopharm. Deionized water was used in all experiments. To remove polymerization inhibitors, all liquid monomers were passed through a basic alumina column before use.

### 2.2 Instrumentation and Characterization

Morphological and elemental analyses were performed using a HITACHI S‐4800 scanning electron microscope (SEM) equipped with energy dispersive spectroscopy (EDS). Moisture absorption kinetics were evaluated in a THL‐6010 M environmental chamber (Shanghai Zhetu Scientific Instrument Corporation) under controlled relative humidity (RH) conditions. Samples were weighed at 30‐minute intervals, and equilibrium water uptake was determined after 6 h of exposure. Photothermal performance was assessed using an HFX‐F300 xenon lamp (NBeT) to simulate sunlight. Humidity and temperature were monitored using an RC‐4HC hygrothermograph (Jiangsu Jingchuang Electric Corporation), while infrared thermal images were captured using a P200 handheld thermal camera (IRay Technology Co., Ltd). Chemical functional groups were analyzed via Fourier transform infrared spectroscopy (FTIR) on a Nicolet iS10 spectrometer (Thermo Fisher Scientific). Thermal stability was evaluated via thermogravimetric analysis (TGA) using a Netzsch TG 209 F3 instrument.

### 2.3 Synthesis of Brush Hydrogel Microsphere

Brush‐type hydrogel microspheres were synthesized via suspension copolymerization of acrylamide (AM) and poly(ethylene glycol) methyl ether methacrylate (PEGMA). The aqueous dispersion phase was prepared by dissolving 1.2 g PEGMA, 0.54 g AM, 0.05 g PEGDA, and 0.088 g KPS in 4 mL of deionized water. A 100‐mL round‐bottom flask fitted with a magnetic stir bar was charged with 20 g cyclohexane (oil phase) and 1 g Span 80 (surfactant). Under mechanical stirring at 500 rpm, the aqueous phase was added dropwise to the oil phase over 10 min. The mixture was then heated to 70°C for 1 h to initiate polymerization. After cooling to room temperature, 30 mL of deionized water was added to extract hydrogel microspheres from cyclohexane. The brush hydrogel microspheres were collected, washed three times with deionized water, and redispersed in water for subsequent use.

### 2.4 Fabrication of Multi‐Layer Heterogeneous Hygroscopic Membrane

A multi‐layer heterogeneous hygroscopic membrane was fabricated via an alternating electrospinning‐spraying process. The electrospinning solution was prepared by dissolving 2 g PAN in 18 g DMF. The solution was loaded into a plastic syringe fitted with a 20 G metal needle and ejected at a flow rate of 0.5 mL h^−1^. Electrospinning was conducted using a 20 cm‐diameter grounded metal plate collector filled with deionized water. A high voltage of +13 kV was applied to the needle, while the collector was maintained at −5 kV. After 30 min of electrospinning, 4 mL of hydrogel microsphere dispersion was uniformly sprayed onto the nanofiber layer. This cycle was repeated twice to form a three‐layer structure (nanofiber/ hydrogel/ nanofiber). The as‐prepared membrane was sequentially washed with deionized water and ethanol to remove impurities. Post‐drying, the membrane was cut into rectangular specimens and immersed in a 10 wt% lithium chloride (LiCl) aqueous solution to facilitate salt loading. Excess solution was drained, and the membrane was dried in an oven at 80°C overnight. The final product was stored in a desiccator before characterization.

### 2.5 Salt Loading of Membranes and Microspheres

Salt loading was performed by immersing dried membranes or hydrogel microspheres in LiCl aqueous solution at the desired concentration. For the MHH membrane, dried specimens were immersed in 10 wt% LiCl solution for 12 h, removed, drained to remove excess solution, and dried at 80°C overnight. For hydrogel microspheres used as controls, the same salt loading protocol or an equivalent mass‐based loading method was applied. Salt content was determined from the mass difference before and after loading and reported as 170 g m^−2^. Samples were stored in a desiccator prior to sorption testing.

### 2.6 Preparation of PAM and Pam‐co‐PEGMA Films

Hydrogel films for permeability measurements were prepared by photopolymerization. For PAM films, 0.76 g AM, 0.05 g PEGDA, and 0.056 g photoinitiator 2959 were dissolved in 4 mL deionized water. For PAM‐co‐PEGMA films, 0.56 g AM, 1.2 g PEGMA, 0.05 g PEGDA, and 0.056 g photoinitiator 2959 were dissolved in 4 mL deionized water. Each solution was transferred into a 4 cm diameter mold, degassed for 10 min, and irradiated with a 365 nm UV lamp (97 mW cm^−^^2^) for 30 min. The resulting films were dried in a convection oven at 60°C for 24 h and stored in a desiccator. Film thickness was measured at 3 positions using a micrometer and reported as the average value.

### 2.7 Water Vapor Permeability Measurements

Water vapor transport through polymer films was measured using a cross‐flow membrane cell. The membrane was sealed at its edges with silicone grease to define an effective transport area of 95 mm^2^. The membrane cell was placed in an environmental chamber maintained at 25°C and 30% RH. Saturated humid air was introduced into the feed chamber at 1 L min^−^^1^, while the humidity in both feed and permeate chambers was monitored continuously using calibrated humidity sensors. Relative humidity values were converted to absolute humidity according to the equation below, and the instantaneous concentration gradient across the membrane was calculated from the humidity difference between the two chambers.

A=C·Pw/T

where A is absolute humidity(g m^−3^), C is a constant (2.16679 gK J^−1^), Pw is vapor pressure in Pa, and T is temperature (K).

The water vapor flux was obtained from the time derivative of the absolute humidity increase in the permeate chamber multiplied by the chamber volume. The apparent diffusion coefficient was then determined from Fick's first law by linear fitting of vapor flux against concentration gradient.

### 2.8 Moisture Absorption, Desorption, and Cycling Tests

Moisture absorption kinetics were measured in a temperature‐ and humidity‐controlled chamber at specified RH values. Dried samples of known mass and dimensions were exposed to humid air at 25°C and the desired RH. Sample mass was recorded at defined time intervals until equilibrium was reached, which was taken as a mass change of less than 0.1 g over 30 min. Water uptake was calculated on a dry‐mass basis. For desorption measurements, saturated samples were irradiated under a xenon lamp at 1 sun while sample mass and temperature were monitored simultaneously. Water retention during desorption was calculated relative to the saturated state. For all sorption/desorption tests, at least 3 independent replicates were measured.

### 2.9 Atmospheric Water Harvesting Tests

Device‐level SAWH performance was evaluated using a three‐bed alternating adsorption/desorption setup. Each bed contained a 5 × 5 cm MHH membrane. Adsorption was carried out in a sealed chamber maintained at 25°C and 90% RH, while desorption was performed in a hemispherical transparent chamber under 1‐sun xenon‐lamp irradiation. The three adsorption beds were alternated between adsorption and desorption with an adsorption time of 2 h and a desorption time of 1 h. Water production was quantified from the mass changes of the sorbent beds and/or condensate collection chamber.

For clarity, the number of harvesting events reported for the three‐bed device refers to sequential bed‐level water‐harvesting operations across the complete system and should not be interpreted as the same number of full adsorption–desorption cycles experienced by each individual membrane bed.

## 3 Results and Discussion

### 3.1 Architectural Design and Structural Validation of a Multi‐Layer Heterogeneous Hygroscopic Membrane

The MHH membrane was designed to separate the functions of vapor capture and liquid storage within a single integrated sorbent. As illustrated in Figure 2a, fabrication involved an alternating electrospinning‐spraying process followed by photothermal coating and salt loading. A porous PAN nanofiber layer was first produced by electrospinning, after which pre‐synthesized brush‐like hydrogel microspheres were sprayed onto the fiber scaffold. A second electrospinning step encapsulated the hydrogel layer, forming a nanofiber/hydrogel/nanofiber sandwich structure. The outer nanofiber layer was then coated with polypyrrole (PPy) through oxidative polymerization and loaded with LiCl to provide hygroscopic functionality [39, 40].

**FIGURE 2:**
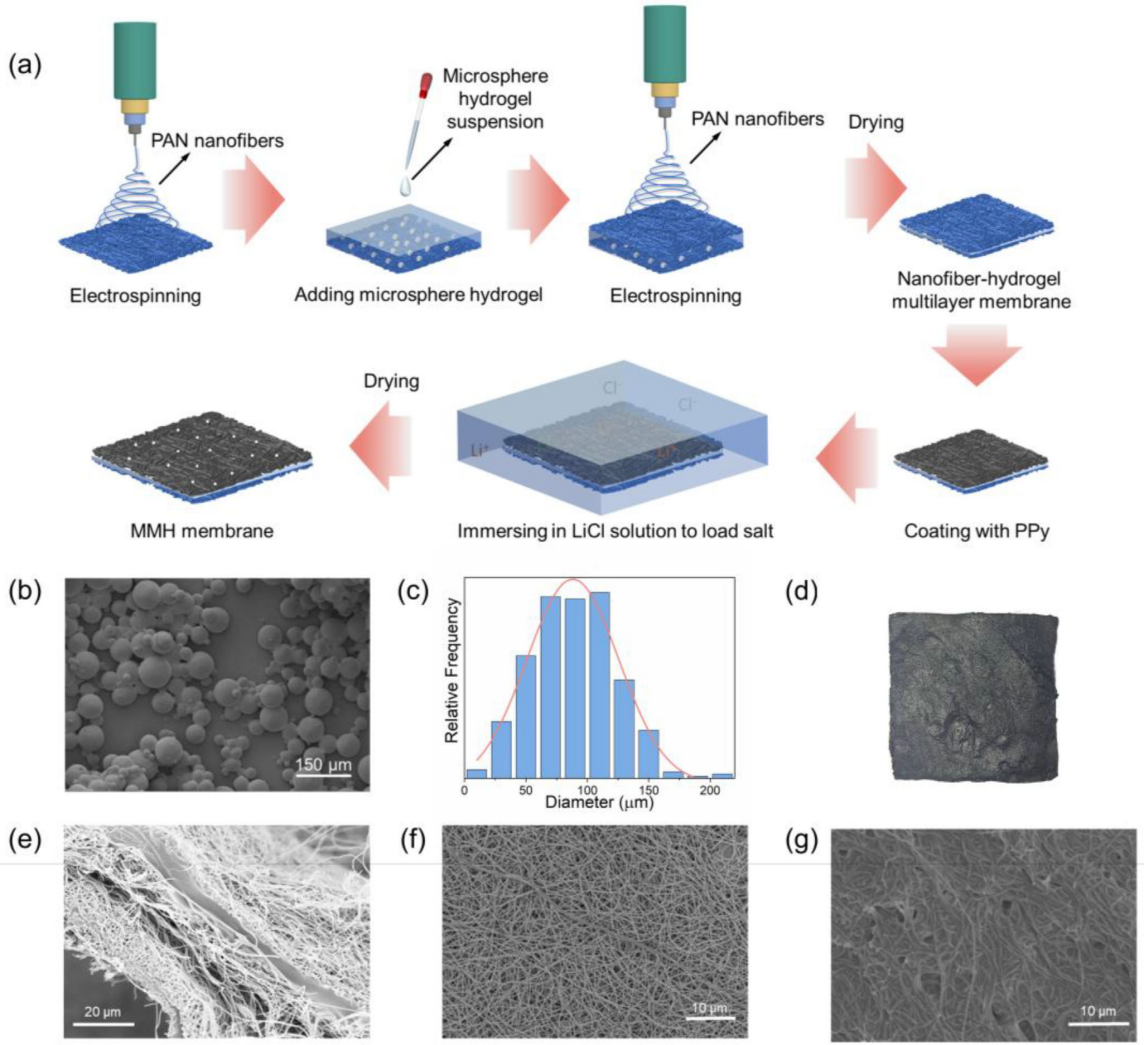
Fabrication and structural characterization of MHH membranes. (a) Schematic of the MHH membrane fabrication process. (b) SEM image of hydrogel microspheres. (c) Diameter distribution of hydrogel microspheres. (d) Sample image of a 5 cm × 5 cm fabricated MHH membrane. (e) Cross‐sectional microstructure images of MHH membranes. (f) SEM image of the nanofiber layer on the surface of the MHH membrane before salt loading. (g) SEM image of the nanofiber layer on the surface of the MHH membrane after salt loading.

Using pre‐formed hydrogel microspheres is important for preserving the intended heterogeneity of the membrane. If hydrogel polymerization occurred directly inside the nanofiber scaffold, the resulting interpenetrating network would obscure phase boundaries and make it difficult to assign distinct roles to the fiber and hydrogel domains. In contrast, the spraying of discrete microspheres allows a hydrogel‐rich interlayer to be formed between two nanofiber layers, creating a defined interface where salt solution can move during wetting and drying cycles.

The morphology of the hydrogel microspheres is shown in Figure 2b. The particles are approximately spherical and have relatively smooth surfaces, indicating successful suspension polymerization. The size distribution obtained from SEM image analysis (Figure 2c) is broad but centered at approximately 100 µm, with the largest particles approaching 200 µm. Although some aggregation is visible after drying, the microspheres are well dispersed in water because swelling provides steric separation (Figure S1). This dispersion stability is necessary for uniform spraying and for avoiding large local defects in the hydrogel interlayer.

The fabricated membrane is shown in Figure 2d. After PPy coating, the membrane becomes black, which is advantageous for solar‐driven desorption. Cross‐sectional SEM imaging (Figure 2e) confirms the multilayer arrangement: a hydrogel‐rich region is located between two nanofiber layers, and the interfaces between these domains remain distinguishable. The hydrogel layer appears continuous because the sprayed microspheres pack closely and partially coalesce during drying. The interfacial contact between the hydrogel and nanofiber domains is sufficient to maintain structural integration, although the precise bonding interactions cannot be assigned from SEM alone. The possible contributions of polar interactions between PEGMA‐containing hydrogel groups and PAN nitrile groups should therefore be regarded as plausible rather than directly proven [41].

Surface SEM images before and after LiCl loading show the intended transformation of the nanofiber interface (Figure 2f,g). Before salt loading, the surface consists of an open and interconnected nanofiber network. After LiCl loading and drying, this fibrous morphology is partly covered by salt deposits, indicating that the inter‐fiber voids can host a substantial amount of hygroscopic salt. This surface‐accessible salt reservoir is central to the proposed function of the membrane because it provides direct vapor‐salt contact at the beginning of adsorption [29].

The salt distribution after drying is expected to be influenced by both the loading step and the redistribution of salt solution during desorption. The increase in salt content with hydrogel loading (Figure S2) indicates that the hydrogel domain contributes strongly to total salt storage, while the surface SEM image after loading (Figure 2g) shows that salt can be retained in the nanofiber layer. As discussed in the next section, EDS and cycling experiments further support surface salt enrichment after repeated wetting and drying. These structural observations establish the physical basis for assigning the nanofiber layer as an air‐accessible capture region and the hydrogel layer as a liquid‐storage reservoir, while leaving the dynamic migration process to be evaluated by sorption and compositional evidence.

### 3.2 Coupled Interfacial Deliquescence and Salt Redistribution for Rapid Moisture Capture

The layered architecture was expected to accelerate sorption by enriching hygroscopic salt at an air‐accessible nanofiber interface while retaining the hydrogel as a storage reservoir. To test this hypothesis, the moisture uptake of the MHH membrane was compared with that of brush‐like hydrogel microspheres with the same substrate/salt mass ratio of 1/2, corresponding to an average salt density of 0.017 g cm^−2^ (Figure S2). Measurements were conducted at 25°C and 70% RH [20, 29].

The two materials reach similar equilibrium water uptake values of approximately 2.4 g g^−1^ (Figure 3a), showing that incorporation of the nanofiber layers does not sacrifice water‐storage capacity. The difference lies mainly in the rate of uptake. The MHH membrane absorbs approximately 1.4 g g^−1^ within the first 30 min, nearly twice the uptake of the microsphere control, and reaches about 85% of its equilibrium capacity within 90 min. The hydrogel microspheres require approximately 300 min to reach a comparable fractional uptake. The differentiated uptake profiles in Figure 3b further show that the initial sorption rate of the MHH membrane is 0.049 g g^−1^ min^−1^, about 2.1 times that of the microspheres (0.023 g g^−1^ min^−1^). Fitting with the unified adsorption model gives a characteristic time constant of 40.12 min for the MHH membrane, compared with 110.72 min for the microspheres (Table S1), confirming the kinetic advantage of the layered structure [29, 42].

**FIGURE 3:**
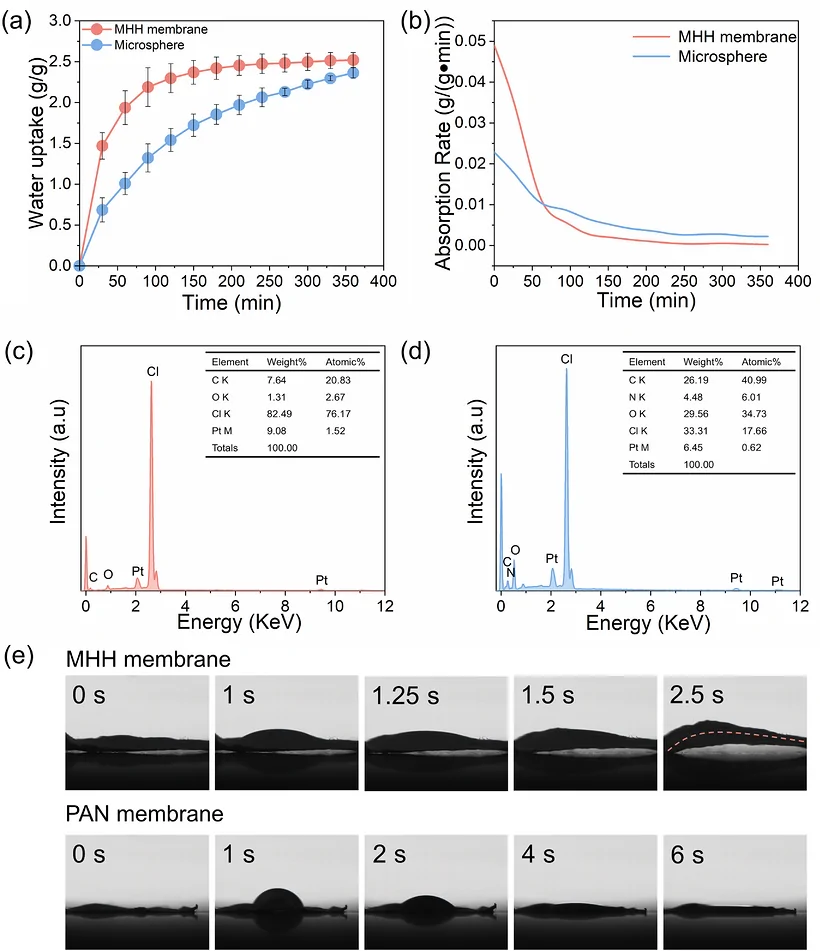
Moisture absorption performance characterization and ‘salt secretion’ mechanism analysis of MHH membranes. (a) Comparison of moisture absorption kinetics between MHH membranes and hydrogel microspheres at 70%RH. (b) Comparison of the moisture absorption rate between MHH membranes and hydrogel microspheres at 70%RH. (c) EDS spectrum and element analysis of the MHH membrane surface. (d) EDS spectrum and element analysis of the hydrogel microsphere surface. (e) Liquid saline aqueous solution absorption experiment of the MHH membrane (up) and the PAN nanofibrous membrane (down).

This kinetic difference is consistent with a change in the early‐stage mass‐transfer pathway. In the MHH membrane, part of the LiCl is located in or near the nanofiber layer after drying, allowing water vapor to contact the salt directly without first diffusing through a dense hydrogel matrix. The initial uptake is therefore dominated by interfacial deliquescence. In the microsphere control, more of the salt is embedded within the polymer particles, so water uptake depends more strongly on transport through the hydrogel phase even at early times. The faster initial uptake of the MHH membrane is therefore best attributed to improved salt accessibility rather than to an increase in total salt capacity [20].

As adsorption continues, the deliquesced salt solution formed at the nanofiber interface must be absorbed into the hydrogel layer. The uptake rate consequently decreases as the interfacial salt reservoir is progressively consumed and liquid transport within the hydrogel becomes more important. This interpretation explains the gradual convergence of the kinetic curves at longer times. It also avoids treating the entire sorption process as a purely interfacial event: the nanofiber layer accelerates the onset of water capture, while the hydrogel layer governs storage and later‐stage transport [20].

The surface distribution of salt was examined by EDS to evaluate whether the nanofiber interface is indeed salt‐enriched. The MHH membrane surface exhibits a Cl/C atomic ratio of 10.80, much higher than the value of 1.27 measured on the hydrogel microsphere surface under the same bulk salt‐loading condition (Figure 3c,d). Because EDS provides static and surface‐weighted compositional information, this result should be interpreted as evidence of surface salt enrichment rather than direct visualization of the migration pathway. Together with the dependence of salt content on hydrogel loading (Figure S2) and the persistence of salt deposits in the nanofiber network after repeated cycles (Figure S6), the data support the proposed salt‐redistribution mechanism during drying [20, 29].

The cross‐sectional microstructure of the MHH membrane was further characterized by SEM to examine the spatial distribution of salt after drying. As shown in Figure S4a, substantial salt deposits occupy the inter‐fiber voids of the outer nanofiber layer, indicating that the air‐accessible nanofiber region serves as an important salt‐enrichment domain after drying. Cross‐sectional SEM‐EDS elemental mapping further distinguishes the PAN‐rich nanofiber regions from the hydrogel interlayer through their characteristic C, N, and O distributions (Figure S4b–f). Cl signals associated with the loaded LiCl are detected across both functional domains, while the pronounced salt deposits within the nanofiber voids provide spatial evidence consistent with redistribution of LiCl toward the nanofiber region during drying. Because these measurements are performed after drying rather than in situ, they do not directly resolve the temporal migration process; instead, together with the surface characterization and reversible cycling results, they support the proposed drying‐induced interfacial salt‐redistribution mechanism [29].

Rapid transfer of deliquesced salt solution from the nanofiber layer into the hydrogel is also required to sustain interfacial vapor capture. This coupling was examined by placing a 10 wt% LiCl aqueous droplet on the membrane surface (Figure 3e). On the MHH membrane, the droplet rapidly collapses and penetrates within about 1.25 s, accompanied by visible local swelling of the membrane. On a pure PAN nanofiber membrane, the droplet remains largely intact for about 6 s. The estimated apparent absorption rate is approximately 32 µL s^−1^for the MHH membrane and 6.7 µL s^−1^ for the PAN membrane. This test is qualitative because droplet geometry and local wetting may influence the calculated rate, but it clearly shows that the hydrogel‐containing membrane provides a much more effective pathway for liquid uptake than the nanofiber scaffold alone.

However, conventional liquid wicking tests primarily characterize bulk liquid uptake and do not directly represent vapor‐induced salt deliquescence under humid operating conditions. To distinguish the kinetic contributions of the sequential mass‐transfer steps involved in moisture capture, we therefore independently evaluated vapor‐triggered deliquescence of LiCl within the PAN nanofiber layer and subsequent uptake of the generated saline solution by the hydrogel layer. LiCl‐loaded PAN nanofiber membranes were used to characterize vapor‐driven salt deliquescence, while standalone hydrogel samples were used to quantify saline‐solution uptake (Figure S5). Under the tested conditions, the measured areal deliquescence rate of the LiCl‐loaded PAN nanofiber layer was approximately 0.0004 g cm^−^^2^ min^−^^1^, whereas the saline‐solution uptake rate of the hydrogel was approximately 0.0014 g cm^−^^2^ min^−^^1^. Although these values were obtained from independent experiments involving different phase‐transfer processes and therefore should not be interpreted as a direct quantitative decomposition of the overall membrane sorption rate, the substantially faster liquid uptake of the hydrogel indicates that it can efficiently accommodate the saline solution generated by interfacial deliquescence. These results suggest that vapor‐driven salt deliquescence is the slower of these two elementary processes under the tested conditions, while the rapid liquid‐transport capability of the hydrogel helps remove the deliquesced solution from the nanofiber region and sustain further vapor capture [20].

The durability of this salt‐enriched interfacial state was examined through repeated adsorption‐desorption cycles. After 20 cycles, the MHH membrane retains a water uptake of approximately 2.5 g g^−1^, and the adsorption curves before and after cycling nearly overlap (Figure 4a). SEM images after cycling show that salt remains in the nanofiber interstices (Figure S6). These results indicate that the surface‐enriched salt configuration can be regenerated during repeated operation and that the enhanced kinetics are not limited to the first loading‐drying cycle.

**FIGURE 4:**
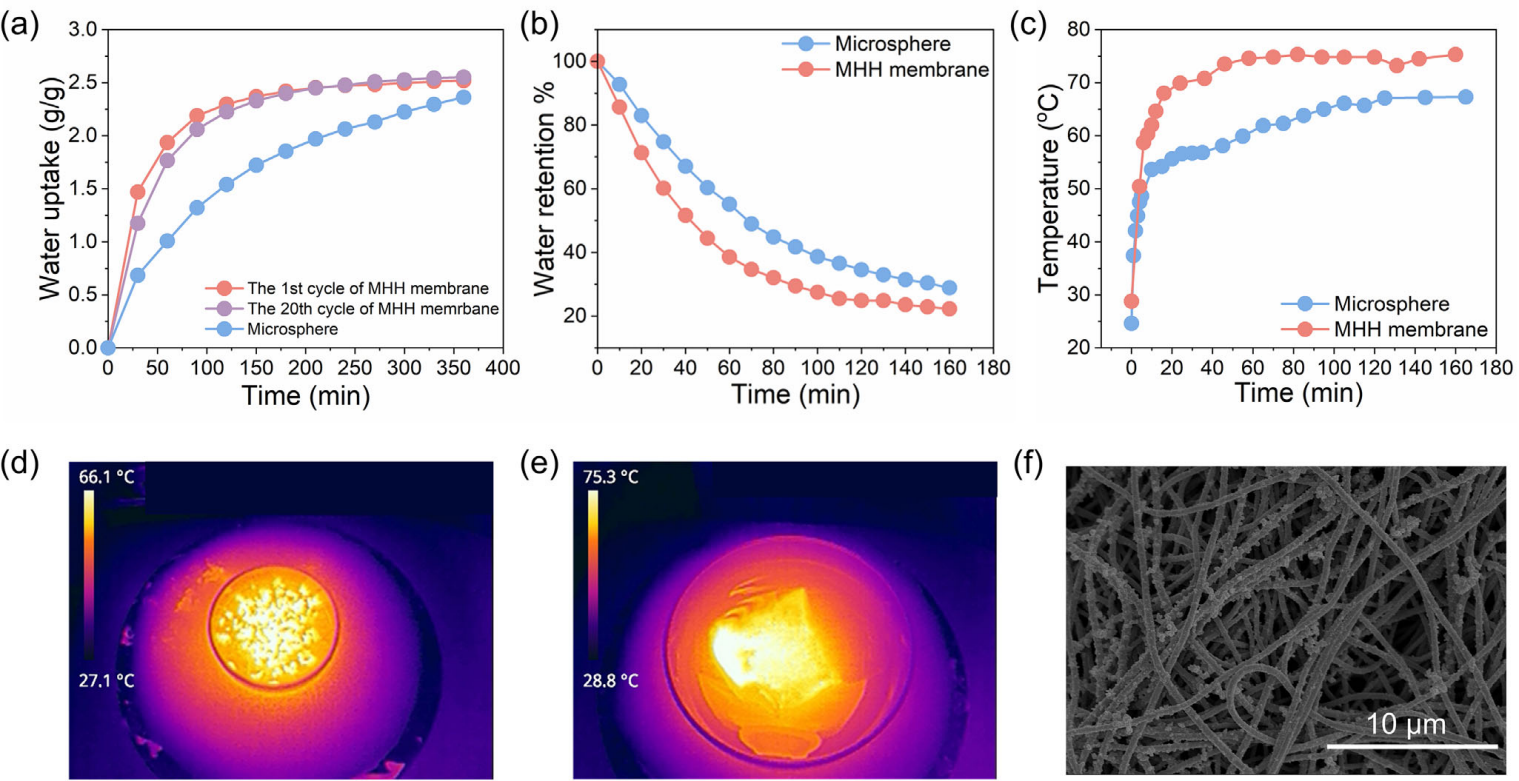
Moisture desorption performance characterization of MHH membranes. (a) Cyclic stability of MHH membranes. (b) Simulated sunlight‐induced desorption of MHH membrane and hydrogel microspheres. (c) Temperature change during the desorption process. (d) Infrared thermal images of the MHH membrane. (e) Infrared thermal images of hydrogel microspheres. (f) SEM image of nanofiber membrane coated with PPy.

The behavior of the MHH membrane under prolonged saturated humidity was further examined by exposing the membrane continuously to 100% RH for 48 h (Figure S7). Visible surface wetting and slight saline‐solution leakage developed during this treatment (Figure S7a), indicating that the present layered architecture does not completely confine the deliquesced salt solution under prolonged saturated conditions. This behavior is consistent with the generation of a substantial volume of deliquesced saline solution that may exceed the storage capability of the hydrogel‐containing membrane under prolonged saturated conditions. After the aged membrane was dried, its moisture‐sorption kinetics were re‐measured (Figure S7b) and remained broadly comparable to those of the untreated membrane, with rapid initial uptake retained during the first 90 min. The post‐aging result indicates recovery of substantial sorption functionality after the saturated‐humidity stress test; however, because the amount of salt lost during leakage was not quantified, these measurements do not establish complete LiCl retention or permanent salt locking. Prolonged saturation therefore represents a boundary condition of the present design and motivates further improvement of liquid‐storage and salt‐confinement capability for operation under extreme humidity.

Desorption under simulated solar irradiation further supports the benefit of the layered configuration. The MHH membrane releases about 70% of its stored water within 90 min, whereas the hydrogel microspheres release about 60% under the same conditions (Figure 4b). The membrane also reaches a higher surface temperature, exceeding 70°C within 20 mins, while the microspheres approach about 60°C (Figure 4c–e). This difference is consistent with the localization of PPy on the outer nanofiber layer (Figure 4f), where it can absorb incident light efficiently and generate heat near the evaporation interface. By contrast, PPy distributed within hydrogel microspheres is more strongly coupled to the hydrated polymer matrix, which increases thermal mass and reduces the effective surface temperature rise [18, 39].

The combined sorption, EDS, droplet‐absorption, cycling, and desorption results support a restrained mechanistic picture. The MHH membrane accelerates early‐stage adsorption because drying leaves hygroscopic salt enriched at an air‐accessible nanofiber interface. The resulting deliquesced solution is then drawn into the hydrogel reservoir, allowing the interface to continue participating in vapor capture. During desorption, surface‐localized PPy and shortened transport pathways facilitate water release and reformation of the salt‐enriched interface. This mechanism directly explains the observed fast cycling without requiring the stronger claim that salt migration has been directly observed in real time.

### 3.3 Brush‐Like Hydrogel Networks Promote Rapid Internal Mass Transport

The interfacial salt‐enrichment mechanism can improve initial vapor capture only if the deliquesced solution can be transported efficiently into the hydrogel reservoir. The hydrogel layer therefore must provide low internal resistance to water and salt‐solution uptake. Conventional PAM hydrogels are attractive because of their hydrophilicity and ease of synthesis, but their transport can be limited by restricted chain mobility, particularly before sufficient swelling has occurred [18].

To reduce this internal resistance, PEG‐based side chains were incorporated into the hydrogel network to form a brush‐like polymer architecture (Figure 5a). The flexible PEG segments were introduced to promote local chain mobility and increase the accessibility of diffusion pathways within the hydrogel. This design does not replace the interfacial salt‐redistribution mechanism; rather, it enables the liquid generated at the nanofiber interface to be accepted rapidly by the bulk hydrogel [36].

**FIGURE 5:**
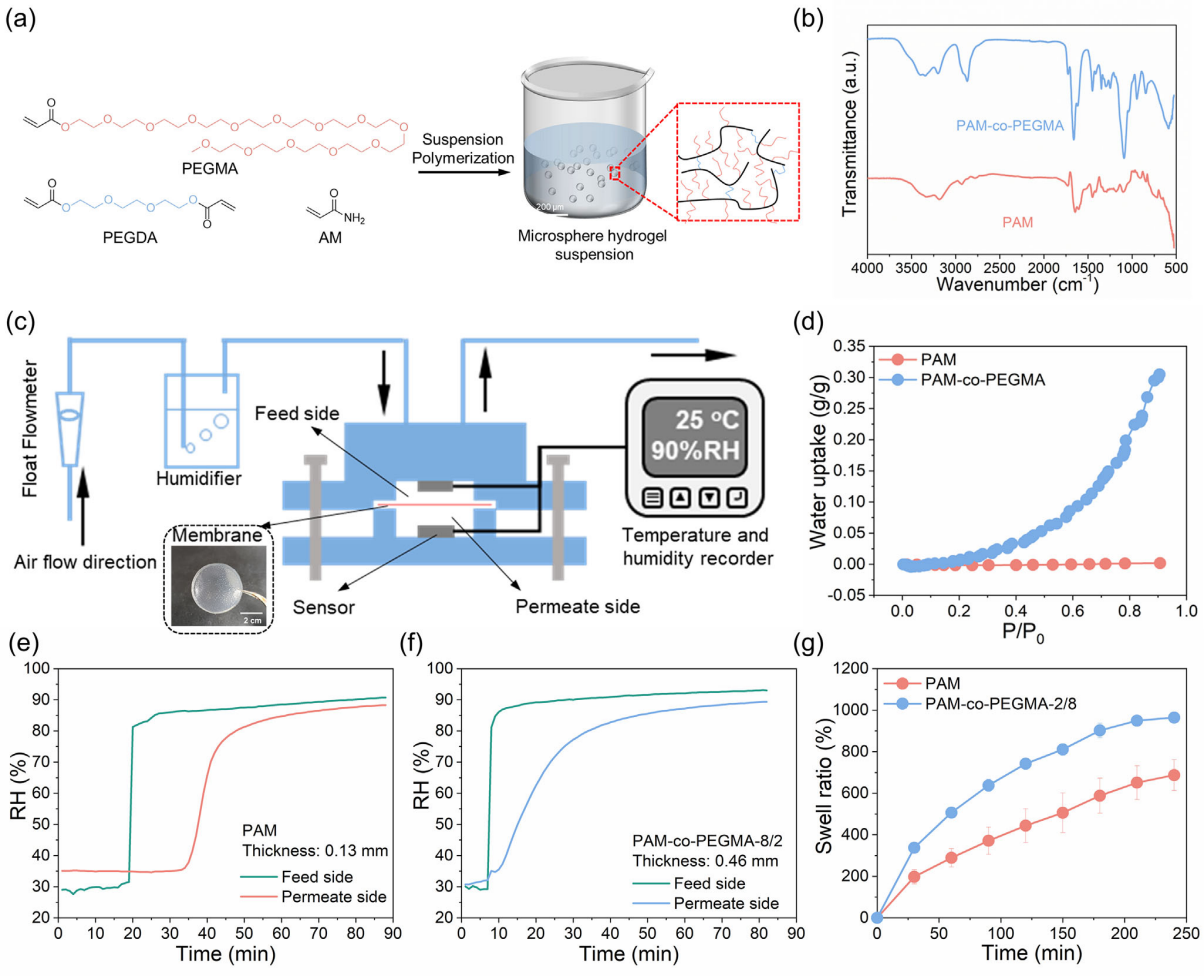
Preparation and performance characterization of brush‐like polymer hydrogel layers. (a) schematic of brush‐like polymer microsphere synthesis. (b) FT‐IR spectra of brush‐like polymer and PAM. (c) schematic of the crossflow membrane cell and vapor permeation experiments. (d) Dynamic vapor sorption results of brush‐like polymer and PAM. (e) Vapor permeation experiment result of the PAM membrane. (f) Vapor permeation experiment result of the brush‐like polymer membrane. (g) Swelling curves in 20% LiCl aqueous solution of brush‐like polymer and PAM.

PEGMA with a number‐average molecular weight (*M_n_*) of 600 Da was selected as an intermediate side‐chain length to provide multiple ethylene‐oxide units for hydration while retaining sufficient segmental flexibility and processability under the ambient‐temperature conditions relevant to atmospheric water harvesting. The transport behavior of hydrated polymer networks is strongly influenced by network structure, solvent–polymer interactions, and polymer‐chain mobility, while PEG methacrylate‐based materials can also exhibit structure‐dependent thermal behavior that affects segmental mobility and transport [36, 38, 39]. Accordingly, the selected *M_n_* =  600 Da PEGMA was used as a practical compromise between hydration capability, chain mobility, and processability in the present brush‐like hydrogel architecture. A systematic comparison of PEGMA molecular weight was not performed in this study; therefore, the present selection should be regarded as a rational design choice rather than an experimentally established global optimum.

FTIR spectra confirm the incorporation of PEG‐containing segments (Figure 5b). Compared with PAM, the brush‐like hydrogel shows a stronger C‐O stretching band at 1087 cm^−1^ associated with ether groups, along with terminal C‐H vibrations at 2866 cm^−1^ from PEG segments [37, 38]. These spectral changes verify the chemical difference between the brush‐like hydrogel and conventional PAM, providing the basis for comparing their transport behavior.

Water‐vapor permeation was measured using a cross‐flow membrane cell (Figure 5c). For the PAM film, the permeate‐side humidity remains nearly unchanged during the initial 15 min after rapid humidification of the feed side (Figure 5e), indicating an appreciable induction period before continuous vapor transport is established. In contrast, the PAM‐co‐PEGMA film exhibits an almost immediate increase in permeate‐side humidity (Figure 5f). Because the two films have different thicknesses, direct comparison of the experimentally observed lag times alone does not provide a fair assessment of their transport behavior. The samples used here are continuous polymer films rather than porous packed beds; therefore, a packing‐density or porosity correction is not applicable to this comparison.

Under the assumptions of the classical diffusion model for a planar film, the characteristic permeation lag time scales with the square of the transport length, (t_lag_∝L^2^), and a time‐lag‐derived effective diffusion coefficient can be estimated from (D_eff_ = L^2^/(6t_lag_) [38]. The PAM film, with an average thickness of 0.14 mm and an experimental lag time of approximately 13 min, corresponds to a lag time of approximately 6.63 min when normalized to a reference thickness of 0.10 mm and gives a time‐lag‐derived effective diffusion coefficient of approximately 4.2 × 10^−8^ cm^2^ s^−^^1^. In comparison, the PAM‐co‐PEGMA film, despite its larger thickness of 0.46 mm, exhibits an experimental lag time of only approximately 2 min, corresponding to a normalized lag time of approximately 0.09 min at the same reference thickness and an effective diffusion coefficient of approximately 2.9 × 10^−6^ cm^2^ s^−^^1^. Thus, the time‐lag‐derived effective diffusivity of PAM‐co‐PEGMA is approximately 70 times that of PAM under the present permeation conditions. Because polymer relaxation contributes to the measured transport response, particularly in PAM, these values are used as comparative effective transport parameters rather than as intrinsic molecular diffusivities [38, 41]. Together with the swelling‐kinetic results discussed below, these observations support substantially faster water transport through the PEG‐containing network and are consistent with reduced polymer‐relaxation‐associated transport resistance [38, 39, 41].

Dynamic vapor sorption measurements show the same trend (Figure 5d). At 25°C, the brush‐like hydrogel exhibits increasing moisture uptake with rising vapor partial pressure, whereas PAM shows negligible uptake within the resolution of the measurement under the same conditions. These data indicate that water molecules can more readily penetrate and interact with the PEG‐containing network. The DVS result should be interpreted as evidence of improved moisture accessibility and transport, rather than as a standalone measurement of free volume.

Swelling kinetics provide an independent comparison of solvent uptake within the hydrogel phase. Fitting the swelling curves using the Alfrey‐type power‐law model (Equation S1) gives an exponent (n) close to 0.5 for PAM‐co‐PEGMA (Figure S8), consistent with predominantly Fickian‐like solvent transport, whereas PAM gives an (n) value closer to 1 (Figure S9), indicating a stronger contribution from polymer‐chain relaxation to the overall swelling process 38,41. In 10 wt% LiCl solution, the PAM‐co‐PEGMA hydrogel reaches a swelling ratio approximately 1.75 times that of PAM within 24 min and approaches approximately 1000% after 240 mins, compared with approximately 600% for PAM (Figure 5g). Together with the vapor‐permeation results, these measurements indicate that incorporation of PEG‐containing side chains substantially reduces relaxation‐associated transport limitations and improves water accessibility within the hydrogel network [38, 39, 41].

The functional relevance of this enhanced swelling behavior is particularly important in concentrated salt solution. Because the MHH membrane generates concentrated LiCl solution at the nanofiber interface during adsorption, rapid accommodation of this liquid by the brush‐like hydrogel can facilitate removal of the deliquesced solution from the interfacial region and thereby help sustain the driving force for continued vapor capture [20, 21].

The effect of brush density was evaluated by varying PEG side‐chain content (Figures S11–S13). Increasing the PEG content from 20 mol% to 40 mol% increases the apparent diffusion coefficient from 3.3 × 10^−5^ to 4.7 × 10^−5^ cm^2^ s^−1^, confirming that higher PEG content can improve transport. However, excessive PEG content causes aggregation of the hydrogel microspheres and poorer dispersion stability (Figure S14), which would compromise the spraying process and membrane uniformity [40]. A PEG content of 20 mol% was therefore selected as a practical balance between transport enhancement and processability. Thermogravimetric analysis further shows that the brush‐like hydrogel remains thermally stable within the operating temperature range of the MHH membrane (Figure S15).

These results define the role of the brush‐like hydrogel in the MHH membrane. The PEG‐containing network does not itself create the interfacial salt reservoir; instead, it reduces internal liquid‐transport resistance once interfacial deliquescence occurs. By combining this hydrogel reservoir with the surface‐enriched nanofiber layer described above, the membrane addresses both major transport steps: fast vapor‐salt contact at the interface and efficient solution uptake into the bulk storage domain.

### 3.4 Cycle‐Accelerated Atmospheric Water Harvesting Enabled by Multi‐Scale Transport Engineering

The practical relevance of the MHH membrane depends on whether its material‐level kinetic advantages translate into higher cycle‐based water production. Moisture uptake was first evaluated under different RH conditions to determine the operating window of the membrane (Figure 6a). Across 50%–90% RH, the membrane maintains rapid initial uptake. At 50% and 70% RH, it reaches more than 85% of equilibrium uptake within 90 min; at 90% RH, it reaches about 70% within the same period. The lower fractional uptake at 90% RH arises because the equilibrium capacity is much higher, so a longer time is needed to approach saturation even though the initial uptake rate remains high [18, 32].

**FIGURE 6:**
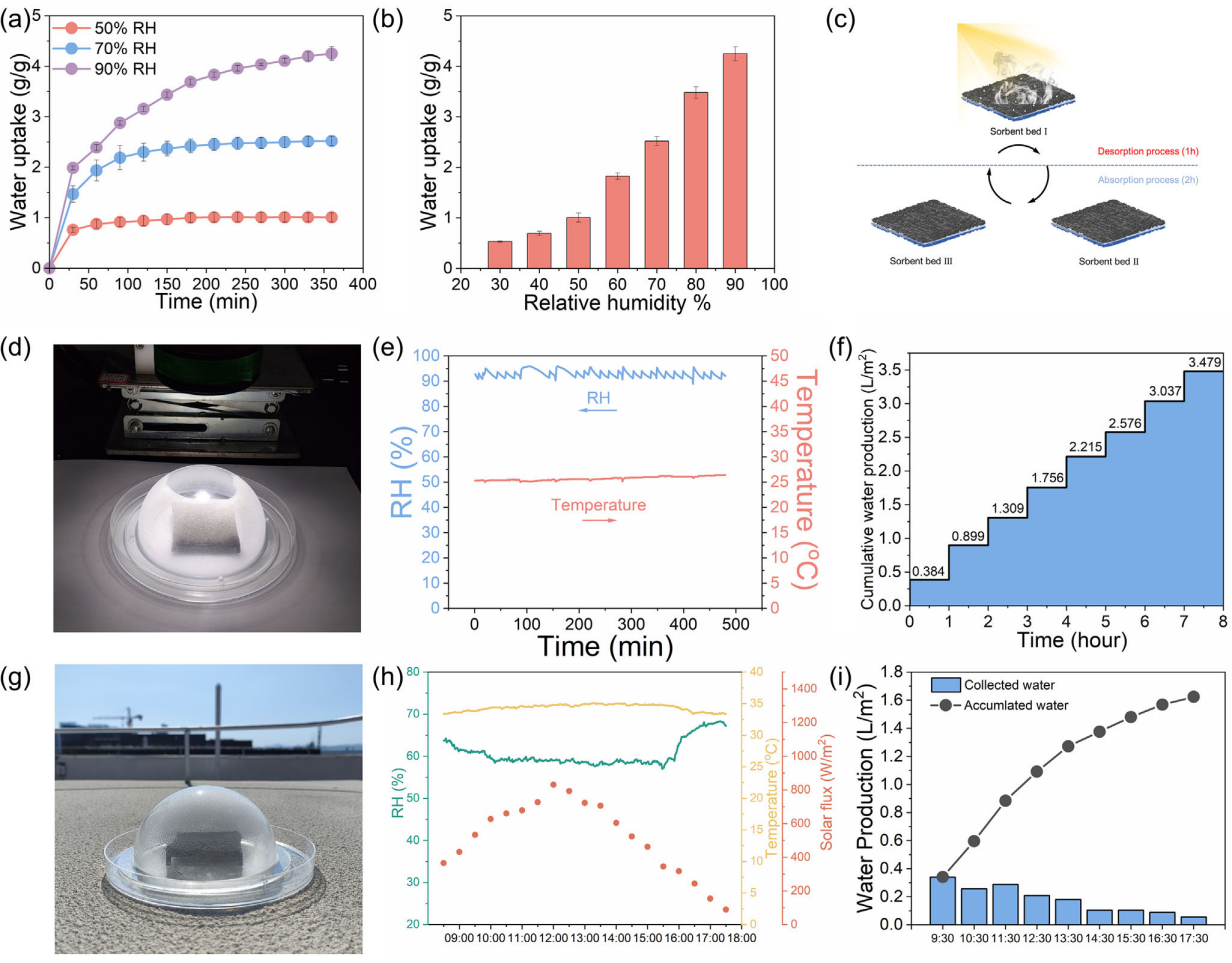
Moisture‐absorbing capability and SAWH performance of MHH membranes. (a) Moisture absorption kinetics of the MHH membrane at different humidity. (b) Maximum water uptake of the MHH membrane under different humidity conditions. (c) Schematic of semi‐continuous SAWH system using MHH membranes. (d) Photo of prototype device in laboratory experiment. (e) Temperature and humidity in the environmental chamber. (f) Water production by semi‐continuous SAWH system using MHH membranes at 90% RH. (g) Photo of prototype device in the outdoor experiment. (h) Ambient temperature, humidity, and sunlight intensity records. (i) Water production of the semi‐continuous SAWH system in one day.

The equilibrium uptake increases monotonically with RH, from approximately 0.5 g g^−1^ at 30% RH to approximately 4.2 g g^−1^ at 90% RH (Figure 6b). This dependence is expected for a LiCl‐containing sorbent because higher vapor activity promotes salt hydration, deliquescence, and formation of concentrated salt solution. The humidity‐dependent data therefore show that the MHH membrane combines high‐capacity operation at high RH with fast early‐stage kinetics across a broad humidity range. The characteristic time constant of 40.12 min obtained from the unified kinetic model (Table S1) further confirms that the membrane is competitive with fast‐cycling hygroscopic polymer sorbents while avoiding freeze‐dried or cryogenically templated porous architectures.

A semi‐continuous three‐bed SAWH device was then constructed to exploit the shortened adsorption‐desorption cycle (Figure 6c). Each bed contained a 5 × 5 cm MHH membrane, and the beds were operated sequentially with 2 h adsorption and 1 h desorption. The device photographs and chamber configuration are shown in Figure 6d and Figures S16 and S17. This arrangement allows one bed to desorb while the others adsorb, thereby converting faster material kinetics into more frequent water‐harvesting cycles [20, 34].

Under controlled laboratory conditions of 25°C and 90% RH (Figure 6e), the device produced 3.47 L m^−2^ over 8 h (Figure 6f), corresponding to an average rate of 0.43 L m^−2^ h^−1^. This high‐humidity benchmark is consistent with the large equilibrium capacity of the membrane at 90% RH and with the accelerated sorption and desorption kinetics demonstrated in Figures 3 and 4. The result should therefore be interpreted as a device‐level demonstration of rapid cycling under favorable humidity, rather than as a universal yield under all climates.

The same prototype was tested outdoors in Xiamen on October 15, 2025, to assess operation under naturally fluctuating conditions (Figure 6g–i). During the test from 08:30 to 17:30, the ambient temperature was approximately 33°C, RH remained around 60%–65%, and peak solar irradiance reached approximately 830 W m^−^^2^ (Figure 6h). Under these conditions, the device achieved an areal water productivity of approximately 1.62 L m^−^^2^ d^−^^1^ over nine sequential bed‐level harvesting events (Figure 6i). The lower water production relative to the controlled laboratory experiment can reasonably be attributed to the combined influence of reduced RH, temporally variable solar irradiation, higher ambient temperature, and less controlled outdoor heat‐, mass‐transfer‐, and condensation conditions. Because these variables change simultaneously during field operation, their individual contributions to the yield difference cannot be quantitatively separated from the present measurements.

A supplementary outdoor field experiment was conducted on August 6, 2026, using the same type of MHH membrane and three‐bed device configuration to evaluate operation under a different set of natural environmental conditions. During this experiment, the ambient RH varied between approximately 63% and 73%, the temperature ranged from approximately 33°C to 37°C, and the maximum solar irradiance reached approximately 1103 W m^−^^2^ (Figure S18a). Intermittent cloud cover resulted in substantial temporal variation in solar irradiation and corresponding fluctuations in the water output of individual harvesting events (Figure S18b). Despite these fluctuations, the device achieved an areal water productivity of approximately 1.964 L m^−^^2^ d^−^^1^.

Because this supplementary field test and the original outdoor experiment were conducted under different meteorological conditions, they should be regarded as independent demonstrations of outdoor operation rather than statistical replicates under identical exposure, and a pooled mean ± SD is therefore not reported. Given the sequential three‐bed operating configuration described above, the reported nine harvesting events refer to sequential bed‐level water‐production events of the complete device and should not be interpreted as nine complete adsorption–desorption cycles experienced by each individual membrane bed.

Water production varies among successive outdoor harvesting events because ambient RH, solar irradiance, temperature, and condensation conditions also vary with time. Consequently, event‐to‐event changes in collected water cannot be attributed solely to material degradation. No obvious monotonic decline in water production was observed during the tested sequence, indicating that repeated water harvesting could be maintained over the duration of the field experiment. However, these measurements do not establish long‐term field durability or statistical reproducibility. Such assessment will require longer‐duration outdoor testing using multiple identical devices operated simultaneously under the same meteorological exposure.

For broader context, the device‐level performance of the MHH membrane was compared with representative recently reported sorption‐based atmospheric water harvesting systems, as summarized in Table S2. The reported systems operate under different relative humidities, temperatures, active sorbent areas, numbers of sorbent beds, cycling strategies, regeneration conditions, condensation configurations, and water‐production reporting bases; therefore, the comparison is intended to provide contextual benchmarking rather than a strict performance ranking. Within this context, the laboratory performance of the present three‐bed device falls within the range of representative recently reported SAWH systems and illustrates how accelerated sorption–desorption kinetics can be translated into repeated water‐harvesting operation.

The quality of the collected condensate is an important consideration for practical atmospheric water harvesting. To evaluate inorganic elemental contamination in the present system, the collected condensate was analyzed using inductively coupled plasma optical emission spectrometry (ICP‐OES). As summarized in Figure S19, the concentrations of the measured elemental species were low and, where applicable, below the corresponding drinking‐water guideline values used for comparison [43]. These results indicate low inorganic elemental contamination of the condensate under the tested conditions. However, ICP‐OES does not assess microbiological contamination, all relevant anionic species, or organic contaminants; these aspects require separate evaluation.

Although the measured inorganic elemental concentrations were low, these measurements alone do not establish the microbiological safety or sterility of the harvested water. PPy is employed in the present MHH membrane primarily as a photothermal coating for solar‐driven regeneration, and no dedicated microbial challenge or sterility measurements were performed for either the membrane or the harvesting device. Microbial contamination may arise not only from the sorbent surface but also from air‐contact interfaces, condenser surfaces, collection vessels, tubing, and subsequent storage. Recent studies have begun to address microbiological safety as an integral component of AWH design. A recent photothermal‐aerogel system combined atmospheric water harvesting with solar‐driven antibacterial activity, demonstrating that the regeneration step can potentially contribute simultaneously to water release and microbial inactivation [44]. In a complementary approach, a biomass‐derived bilayer aerogel with intrinsic antibacterial functionality was developed to improve the microbial safety of harvested water [45]. These studies illustrate two material‐level strategies—regeneration‐associated bacterial inactivation and intrinsic antibacterial sorbent design—for reducing microbial risk. Nevertheless, microbiological safety remains a system‐level issue because contamination can also be introduced downstream during vapor condensation, water collection, transfer, and storage. Practical potable‐water production therefore requires not only appropriate sorbent design but also hygienic condenser and collection components, microbial monitoring, and, where necessary, post‐collection disinfection. Accordingly, the present ICP‐OES results should be interpreted specifically as an assessment of inorganic elemental contamination under the tested conditions rather than as verification of microbiological sterility or complete potability.

The device‐level performance therefore reflects the complementary transport mechanisms established above, in which accelerated interfacial vapor capture, efficient liquid uptake, and photothermal regeneration collectively shorten the effective adsorption–desorption cycle [20, 21]. Further device‐level improvements should focus particularly on condenser efficiency, heat localization, airflow management, and recovery of desorbed vapor.

## 4 Conclusion

A biomimetic MHH membrane has been developed to address the long‐standing capacity–kinetics trade‐off in SAWH. By spatially separating vapor capture from bulk liquid storage, the membrane establishes an air‐accessible nanofiber interface for rapid interfacial deliquescence while retaining a brush‐like hydrogel reservoir for efficient liquid uptake and storage. This architecture enables hygroscopic salt enrichment at the nanofiber interface during drying, thereby reducing the dependence of early‐stage moisture capture on slow diffusion through dense hydrogel matrices. Simultaneously, PEG‐containing side chains enhance internal mass transport by alleviating polymer‐relaxation limitations and facilitating rapid absorption of deliquesced salt solution into the hydrogel domain. The combined interfacial and molecular transport engineering results in substantially accelerated sorption–desorption kinetics while preserving high equilibrium water uptake. Importantly, the material‐level transport advantages are successfully translated into a semi‐continuous device capable of stable atmospheric water harvesting under high‐humidity conditions. Beyond the specific membrane demonstrated here, this work establishes a general design principle in which interfacial accessibility and internal transport are engineered cooperatively across multiple length scales. Such a strategy may provide broader opportunities for advancing hygroscopic sorbents, soft transport materials, solar‐driven separation systems, and interfacial mass‐transfer technologies. Future development of this concept toward adaptive salt chemistries, scalable membrane manufacturing, and low‐humidity operation could further accelerate the deployment of energy‐efficient decentralized water‐harvesting systems.

## Author Contributions

**Fu‐Yao Zhong**: conceptualization, investigation, writing – original draft, methodology, visualization, formal analysis, data curation. **Chao‐Yang Guo**: methodology, software, data curation, formal analysis, investigation. **Zai‐Dong Shao**: conceptualization, methodology, data curation, formal analysis, writing – review and editing. **Lu‐Bing Zhong**: conceptualization, investigation, project administration, data curation, validation. **George Zheng Chen**: writing – review and editing, supervision, conceptualization, project administration. **Yaoyao Zheng**: resources, writing – review and editing, methodology, investigation. **John L. Zhou**: resources, writing – review and editing, investigation. **Yu‐Ming Zheng**: conceptualization, writing – review and editing, supervision, resources, funding acquisition, investigation. **Jun He**: conceptualization, funding acquisition, writing – review and editing, supervision, resources, project administration.

## Conflicts of Interest

The authors declare no conflicts of interest.

## Supporting information




**Supporting File**: advs78196‐sup‐0001‐SuppMat.docx.

## Data Availability

The data that support the findings of this study are available from the corresponding author upon reasonable request.

## References

[1] P. Poredoš , H. Shan , C. Wang , F. Deng , and R. Wang , “Sustainable Water Generation: Grand Challenges in Continuous Atmospheric Water Harvesting,” Energy & Environmental Science 15, no. 8 (2022): 3223–3235, 10.1039/D2EE01234K.

[2] J. Wang , L. Hua , C. Li , and R. Wang , “Atmospheric Water Harvesting: Critical Metrics and Challenges,” Energy & Environmental Science 15, no. 12 (2022): 4867–4871, 10.1039/D2EE03079A.

[3] C. Zhang , H. Guo , C. Li , et al., “Atmospheric Water Extraction—A Review From Materials To Devices,” Journal of Materials Chemistry A 11, no. 41 (2023): 22041–22057, 10.1039/D3TA04380K.

[4] R. Peeters , H. Vanderschaeghe , J. Rongé , and J. A. Martens , “Fresh Water Production From Atmospheric Air: Technology And Innovation Outlook,” iScience 24 (2021): 103266. 10.1016/j.isci.2021.103266 PMC8567397 34761186

[5] A. Entezari , O. C. Esan , X. Yan , R. Wang , and L. An , “Sorption‐Based Atmospheric Water Harvesting: Materials, Components, Systems, and Applications,” Advanced Materials 35 (2023): 2210957. 10.1002/adma.202210957 36869587

[6] C. Liu , X. Y. Yan , S. Li , et al., “A Metre‐scale Vertical Origami Hydrogel Panel for Atmospheric Water Harvesting in Death Valley,” Nature Water 3, no. 6 (2025): 714–722, 10.1038/s44221-025-00447-2.

[7] R. Li , Y. Shi , M. Wu , S. Hong , and P. Wang , “Photovoltaic Panel Cooling By Atmospheric Water Sorption–Evaporation Cycle,” Nature Sustainability 3, no. 8 (2020): 636–643, 10.1038/s41893-020-0535-4.

[8] X. Min , Z. Wu , T. Wei , et al., “High‐Yield Atmospheric Water Harvesting Device With Integrated Heating/Cooling Enabled by Thermally Tailored Hydrogel Sorbent,” ACS Energy Letters 8, no. 7 (2023): 3147–3153, 10.1021/acsenergylett.3c00682.

[9] J. Li , C. Xu , L. Chen , X. Zhang , M. Zhu , and Y. Cheng , “Tandem Atmospheric Water Harvesting and Passive Cooling Enabled by Hygroscopic Biopolymer‐Based Aerogels,” Advanced Functional Materials 35, no. 31 (2025): 2423063, 10.1002/adfm.202423063.

[10] H. Shan , P. Poredoš , Z. Ye , et al., “All‐Day Multicyclic Atmospheric Water Harvesting Enabled by Polyelectrolyte Hydrogel With Hybrid Desorption Mode,” Advanced Materials 35, no. 35 (2023): 2302038, 10.1002/adma.202302038. 37199373

[11] H. Zhou , L. Yan , D. Tang , et al., “Solar‐Driven Drum‐Type Atmospheric Water Harvester Based on Bio‐Based Gels With Fast Adsorption/Desorption Kinetics,” Advanced Materials 36, no. 32 (2024): 2403876, 10.1002/adma.202403876. 38739951

[12] J. Yang , X. Zhang , H. Qu , et al., “A Moisture‐Hungry Copper Complex Harvesting Air Moisture for Potable Water and Autonomous Urban Agriculture,” Advanced Materials 32, no. 39 (2020): 2002936, 10.1002/adma.202002936. 32743963

[13] J. Wang , Q. Shi , H. Shan , et al., “Natural Fibers Enable Textile‐based Composite Sorbent for all‐day Atmospheric Water Harvesting,” Advanced Fiber Materials 8, no. 5 (2026): 1930–1941, 10.1007/s42765-026-00715-0.

[14] D. K. Nandakumar , Y. Zhang , S. K. Ravi , N. Guo , C. Zhang , and S. C. Tan , “Solar Energy Triggered Clean Water Harvesting from Humid Air Existing above Sea Surface Enabled by a Hydrogel with Ultrahigh Hygroscopicity,” Advanced Materials 31 (2019): 1806730. 10.1002/adma.201806730 30637806

[15] H. Shan , P. Poredoš , Z. Chen , et al., “Hygroscopic Salt‐embedded Composite Materials for Sorption‐based Atmospheric Water Harvesting,” Nature Reviews Materials 9, no. 10 (2024): 699–721, 10.1038/s41578-024-00721-x.

[16] T. Lyu , Y. Han , Z. Chen , X. Fan , and Y. Tian , “Hydrogels and Hydrogel Derivatives for Atmospheric Water Harvesting,” Materials Today Sustainability 25 (2024): 100693, 10.1016/j.mtsust.2024.100693.

[17] H. Abdulbakee Muhammed , M. Shahadat , S. A. Tweib , et al., “Harvesting of Atmospheric Water Using Polymer‐Based Hybrid Hydrogels,” ChemBioEng Reviews 11, no. 2 (2024): 197–214, 10.1002/cben.202300032.

[18] F. Ni , P. Xiao , C. Zhang , and T. Chen , “Hygroscopic Polymer Gels Toward Atmospheric Moisture Exploitations for Energy Management and Freshwater Generation,” Matter 5, no. 9 (2022): 2624–2658, 10.1016/j.matt.2022.06.010.

[19] Z. Zhang , H. Fu , Z. Li , et al., “Hydrogel Materials for Sustainable Water Resources Harvesting & Treatment: Synthesis, Mechanism and Applications,” Chemical Engineering Journal 439 (2022): 135756, 10.1016/j.cej.2022.135756.

[20] C. D. Díaz‐Marín , L. Zhang , Z. Lu , M. Alshrah , J. C. Grossman , and E. N. Wang , “Kinetics of Sorption in Hygroscopic Hydrogels,” Nano Letters 22 (2022): 1100–1107. 10.1021/acs.nanolett.1c04216 35061401

[21] C. D. Díaz‐Marín , L. Zhang , B. E. Fil , et al., “Heat and Mass Transfer in Hygroscopic Hydrogels,” International Journal of Heat and Mass Transfer 195 (2022): 123103.

[22] K. Yang , T. Pan , Q. Lei , X. Dong , Q. Cheng , and Y. Han , “A Roadmap to Sorption‐Based Atmospheric Water Harvesting: From Molecular Sorption Mechanism to Sorbent Design and System Optimization,” Environmental Science & Technology 55, no. 10 (2021): 6542–6560, 10.1021/acs.est.1c00257. 33914502

[23] F. Deng , Z. Chen , C. Wang , C. Xiang , P. Poredoš , and R. Wang , “Hygroscopic Porous Polymer for Sorption‐Based Atmospheric Water Harvesting,” Advanced Science 9, no. 33 (2022): 2204724, 10.1002/advs.202204724. PMC9685462 36209387

[24] J. Wang , W. Ying , B. Lin , et al., “Tillandsia‐Inspired Ultra‐Efficient Thermo‐Responsive Hygroscopic Nanofibers for Solar‐Driven Atmospheric Water Harvesting,” Advanced Materials 37, no. 3 (2025): 2408977, 10.1002/adma.202408977. 39478638

[25] X. Zhang , H. Qu , X. Li , et al., “Autonomous Atmospheric Water Harvesting Over a Wide RH Range Enabled by Super Hygroscopic Composite Aerogels,” Advanced Materials 36, no. 41 (2024): 2310219, 10.1002/adma.202310219. 38219071

[26] R. Li , Y. Shi , M. Alsaedi , M. Wu , L. Shi , and P. Wang , “Hybrid Hydrogel With High Water Vapor Harvesting Capacity for Deployable Solar‐Driven Atmospheric Water Generator,” Environmental Science & Technology 52, no. 19 (2018): 11367–11377, 10.1021/acs.est.8b02852. 30192516

[27] J. Xu , T. Li , T. Yan , et al., “Ultrahigh Solar‐driven Atmospheric Water Production Enabled by Scalable Rapid‐cycling Water Harvester With Vertically Aligned Nanocomposite Sorbent,” Energy & Environmental Science 14, no. 11 (2021): 5979–5994, 10.1039/D1EE01723C.

[28] H. Zou , J. Zhu , X. Yang , et al., “High‐Efficiency Atmospheric Water Harvesting and Irrigation Recycling in Greenhouse Using Hygroscopic Composite Gels,” Advanced Functional Materials 35, no. 31 (2025): 2501163, 10.1002/adfm.202501163.

[29] T. Li , T. Yan , P. Wang , et al., “Scalable and Efficient Solar‐driven Atmospheric Water Harvesting Enabled by Bidirectionally Aligned and Hierarchically Structured Nanocomposites,” Nature Water 1, no. 11 (2023): 971–981, 10.1038/s44221-023-00150-0.

[30] J. Sun , F. Ni , J. Gu , et al., “Entangled Mesh Hydrogels With Macroporous Topologies via Cryogelation for Rapid Atmospheric Water Harvesting,” Advanced Materials 36, no. 27 (2024): 2314175, 10.1002/adma.202314175. 38635920

[31] Y. Zhao , W. Guan , Y. Z. Wong , et al., “Material‐to‐system Tailored Multilayer‐cyclic Strategy Toward Practical Atmospheric Water Harvesting,” Proceedings of the National Academy of Sciences 122, no. 20 (2025): 2500928122, 10.1073/pnas.2500928122. PMC12107120 40354535

[32] A. Entezari , M. Ejeian , and R. Wang , “Super Atmospheric Water Harvesting Hydrogel With Alginate Chains Modified With Binary Salts,” ACS Materials Letters 2, no. 5 (2020): 471–477, 10.1021/acsmaterialslett.9b00315.

[33] J. Lu , J. Yan , F. Pei , et al., “Shrinkage‐Resistant Thermo‐Responsive Hygroscopic Hydrogel Toward Ultra‐Rapid Cycling Atmospheric Water Harvesting,” Advanced Functional Materials 35, no. 42 (2025): 2505359, 10.1002/adfm.202505359.

[34] X. Yang , Z. Chen , C. Xiang , H. Shan , and R. Wang , “Enhanced Continuous Atmospheric Water Harvesting With Scalable Hygroscopic Gel Driven by Natural Sunlight and Wind,” Nature Communications 15, no. 1 (2024): 7678, 10.1038/s41467-024-52137-4. PMC11372108 39227366

[35] C. Fu , D. Zhan , G. Tian , A. Yu , L. Yao , and Z. Guo , “Biomimetic Aerogel Composite for Atmospheric Water Harvesting,” ACS Applied Materials & Interfaces 16, no. 27 (2024): 35740–35751, 10.1021/acsami.4c05041. 38918074

[36] T. Fujiyabu , X. Li , U. Chung , and T. Sakai , “Diffusion Behavior of Water Molecules in Hydrogels With Controlled Network Structure,” Macromolecules 52, no. 5 (2019): 1923–1929, 10.1021/acs.macromol.8b02488.

[37] R. E. Coopman , H. T. Nguyen , M. Mencuccini , et al., “Harvesting Water From Unsaturated Atmospheres: Deliquescence Of Salt Secreted Onto Leaf Surfaces Drives Reverse Sap Flow In A Dominant Arid Climate Mangrove, Avicennia marina,” New Phytologist 231, no. 4 (2021): 1401–1414, 10.1111/nph.17461. 33983649

[38] L. Masaro and X. X. Zhu , “Physical Models of Diffusion for Polymer Solutions, Gels and Solids,” Progress in Polymer Science 24, no. 5 (1999): 731–775, 10.1016/S0079-6700(99)00016-7.

[39] G. Guzmán , D. P. Nava , J. Vazquez‐Arenas , and J. Cardoso , “Design of a Zwitterion Polymer Electrolyte Based on Poly[poly (ethylene glycol) methacrylate]: The Effect of Sulfobetaine Group on Thermal Properties and Ionic Conduction,” Macromolecular Symposia 374, no. 1 (2017): 1600136, 10.1002/masy.201600136.

[40] L. H. Gaabour , “Spectroscopic and Thermal Analysis Of Polyacrylamide/Chitosan (PAM/CS) Blend Loaded By Gold Nanoparticles,” Results in Physics 7 (2017): 2153–2158.

[41] P. Lyu , Z. Ding , M. Doi , and X. Man , “A Unified Model for Non‐Fickian Diffusion and Anomalous Swelling of Glassy Polymer Gels,” ACS Macro Letters 13, no. 5 (2024): 483–488, 10.1021/acsmacrolett.4c00041. 38592729

[42] F. Zhao , X. Zhou , Y. Liu , Y. Shi , Y. Dai , and G. Yu , “Super Moisture‐Absorbent Gels for All‐Weather Atmospheric Water Harvesting,” Advanced Materials 31, no. 10 (2019): 1806446, 10.1002/adma.201806446. 30633394

[43] Y. Guan , D. Tang , X. Yu , et al., “Scalable Atmospheric Water Harvesting Paper Enable Rapid and Continuous Water Collection,” Advanced Energy Materials 16, no. 20 (2026): 70889, 10.1002/aenm.70889.

[44] F. Cheng , H. Li , Z. Wei , et al., “Pathogen‐free Atmospheric Water Harvesting Using a Mussel‐inspired Wet‐Adhesive Photothermal Aerogel,” Nature Water 4, no. 3 (2026): 348–359, 10.1038/s44221-026-00592-2.

[45] Z. Duan , P. Feng , M. Ding , C. Y. Liu , C. Li , and J. Zhang , “Biomass‐Derived Bilayer Aerogels for Continuous All‐Day Atmospheric Water Harvesting: Synergistic Adsorption‐Desorption and Antimicrobial Applications,” Advanced Functional Materials 36, no. 2 (2026): 11143, 10.1002/adfm.202511143.

