# Supplementary material for: Interfacial Salt Redistribution Enables Rapid Atmospheric Water Harvesting in a Biomimetic Multi‐Layer Hygroscopic Membrane
Source: Adv Sci (Weinh). 2026 Oct 6:e78196. Online ahead of print. doi: 10.1002/advs.78196 (PMC13643102; doi:10.1002/advs.78196)
Supplement: Supplementary file 1 [file ADVS-9999-e78196-s001.docx]

**Interfacial salt redistribution enables rapid atmospheric water harvesting in a biomimetic multi-layer hygroscopic membrane**

Fu-Yao Zhong ^1,2^, Chao-Yang Guo ^1^, Zai-Dong Shao ^1^, Lu-Bing Zhong ^1^, George Zheng Chen ^3*^, Yaoyao Zheng ^4^, John L. Zhou ^5^, Yu-Ming Zheng ^1^*, Jun He ^2^*

^1^ State Key Laboratory of Advanced Environmental Technology, Institute of Urban Environment, Chinese Academy of Sciences, Xiamen, Fujian 361021, China

^2^ Department of Chemical and Environmental Engineering, Climate and Environmental Solution Research Center, University of Nottingham Ningbo China, Ningbo, Zhejiang 315100, China

^3^ Department of Chemical and Environmental Engineering, University of Nottingham, Nottingham NG7 2RD, UK

^4^ Faculty of Engineering, University of Nottingham, Nottingham NG7 2RD, UK

^5^ Faculty of Science and Engineering, University of Nottingham Ningbo China, China

*Correspondence authors:

Prof. Jun He, email: jun.he@nottingham.edu.cn

Prof. Yuming Zheng, email: [ymzheng@iue.ac.cn](mailto:ymzheng@iue.ac.cn)

Prof. George Chen, email: <george.chen@nottingham.ac.uk>

# 1. Salt content of MHH membranes

MHH membranes with varying hydrogel microsphere contents were prepared by controlling the ratio of the nanofiber layer to the hydrogel layer. Subsequently, the prepared MHH membranes were immersed in a lithium chloride solution of 20% concentration for 3 hours, and the salt content of each membrane was calculated based on mass variation. The pure PAN nanofiber membrane showed the lowest salt content, whereas the salt content of MHH membranes was positively correlated with hydrogel loading.

Figure S1. Photo of microsphere hydrogel dispersion

Figure S2. Salt loading of different MHH membranes.

Figure S3. Photo of dry MHH membrane.

In the below Figure S4, for cross-sectional SEM/EDS characterization, dried MHH membranes were cryo-fractured to expose the internal multilayer cross-section prior to SEM imaging and elemental mapping.

Figure S4. (a) Cross-sectional SEM image of MHH membrane. Cross-sectional SEM-EDS elemental mapping results of original image (b), Cl element (c), C element (d), N element (e), and O element (f).

For Figure S4, the vapor-driven deliquescence kinetics were measured using LiCl-loaded PAN nanofiber membranes, while the saline-liquid uptake kinetics were measured independently using standalone PAM-co-PEGMA hydrogel samples exposed to LiCl solution. The sample area, LiCl loading, and time interval used to determine the areal rates were kept fixed for each respective measurement. The corresponding rates were calculated from the initial approximately linear region of the uptake curves.

Figure S5. Comparison of deliquescence rate and liquid absorption rate.

Figure S6. Salt deposition status of MHH membranes after multiple cycles.

For Figure S7, to assess salt confinement under an extreme humidity condition, an MHH membrane was continuously exposed to 100% RH for 48 h. Visible surface wetting and slight saline-solution leakage developed during exposure, demonstrating that complete LiCl confinement is not maintained under prolonged saturation. After the sample was dried, its moisture-sorption kinetics were remeasured to assess functional recovery. The aged membrane retained rapid initial sorption behavior; however, because salt loss was not quantified, this result should not be interpreted as evidence of complete salt retention.

Figure S7. Saturated-humidity stress test of the MHH membrane. (a) Appearance of the membrane after continuous exposure to 100% RH for 48 h, showing surface wetting and slight saline-solution leakage. (b) Moisture-sorption kinetics after drying of the aged membrane.

# 2. The theory proposed by Alfrey et al

The swelling behavior of polymer networks can be interpreted by considering the relative rates of solvent diffusion and polymer-chain relaxation, which give rise to Fickian and non-Fickian transport regimes^1^. The swelling curves were therefore fitted using the following power-law relationship:

The coefficient n typically ranges from 0.5 to 1.

$\frac{M_{t}}{M_{\infty}}=kt^{n}$	Eq.S1

where M_t_ is the mass of swelled polymeric gel at the t moment. M_∞_ is the mass of swelled polymeric gel at the end of the swelling process, k and n are constants.

The closer n is to 0.5, the higher the relaxation rate of polymer chains relative to the diffusion rate of the solvent. In this case, the diffusion behavior of the solvent within the polymer is closer to Fickian diffusion. Generally, such diffusion behavior occurs when the polymer is in the rubbery state.

The closer n is to 1, the higher the diffusion rate of the solvent relative to the relaxation rate of polymer chains, and the corresponding diffusion behavior is closer to non-Fickian diffusion. Typically, this type of diffusion behavior is observed when the polymer is in the glassy state.

Figure S8. The fitting curve of the PAM-co-PEGMA swelling experiment.

Figure S9. The fitting curve of the PAM swelling experiment.

# 3. Thickness-Normalized Permeation Lag Time and Time-Lag-Derived Effective Diffusivity

Because the PAM and PAM-co-PEGMA films used for vapor-permeation measurements have different thicknesses, the experimentally observed permeation lag times were corrected for transport length before comparison. For a planar film under the assumptions of the classical time-lag diffusion model, the characteristic lag time is related to film thickness and effective diffusivity by ^1^:

$t_{\mathrm{lag}}=\frac{L^{2}}{6D_{\mathrm{eff}}}$	Eq S2

where $t_{\mathrm{lag}}$ is the experimentally measured permeation lag time, $L$ is the film thickness, and $D_{\mathrm{eff}}$ is the time-lag-derived effective diffusion coefficient .

The measured PAM film thickness was approximately 0.14 mm and its permeation lag time was approximately 13 mins. Normalization to a common reference thickness of 0.10 mm gives a lag time of approximately 6.63 min, corresponding to:

$D_{\mathrm{eff}}=\frac{L^{2}}{6\text{ }t_{\mathrm{lag}}}=\frac{\left( 0.010\text{ cm})^{2} \right.}{6\times(6.63\times60\text{ s})}\approx4.2\times{10}^{-8}\text{ }\text{cm}^{2}\text{s}^{-1}$	Eq S3

The PAM-co-PEGMA film had a thickness of approximately 0.46 mm and a measured lag time of approximately 2 min. Its corresponding normalized lag time at 0.10 mm is approximately 0.09 min, giving:

$D_{\mathrm{eff}}=\frac{L^{2}}{6\text{ }t_{\mathrm{lag}}}=\frac{\left( 0.010\text{ cm})^{2} \right.}{6\times(0.09\times60\text{ s})}\approx2.9\times{10}^{-6}\text{ }\text{cm}^{2}\text{s}^{-1}$	Eq S4

Accordingly, the time-lag-derived effective diffusivity of PAM-co-PEGMA is approximately 70 times that of PAM under the present experimental conditions. Because swelling and polymer-chain relaxation can contribute to the measured permeation response, particularly for PAM, these values are interpreted as comparative effective transport coefficients rather than intrinsic molecular diffusion coefficients ^1^.

The films used in these measurements were continuous polymer films rather than porous packed beds. Consequently, a separate packing-density correction is not applicable. The time-lag-derived effective diffusivities reported here should also be distinguished from the apparent diffusion coefficients obtained below from the independent flux–concentration-gradient analysis based on Fick's first law, because the two parameters are extracted using different analytical procedures.

# 4. **Flux-gradient-derived apparent diffusion coefficient**

Figure S10. Humidity change during PAM-co-PEGMA-6/4 permeation experiment.

The calculation method for the apparent diffusion coefficient is illustrated in Figure S10. As a complementary transport analysis, apparent diffusion coefficients were also estimated independently from the relationship between measured vapor flux and instantaneous concentration gradient according to Fick’s first law.

Equation S3 serves to calculate the water vapor flux through the membrane at each instant. This value is primarily derived by differentiating the growth curve of the absolute humidity on the permeate side and subsequently multiplying by the chamber volume. Finally, in accordance with Fick's First Law, linear fitting is conducted on the concentration gradient and water vapor flux at each instant. The apparent diffusion coefficient is thus obtained by dividing the slope of the fitting line by the area.

$\frac{dC}{dX}=\frac{Humdity\left( feed side \right)-Humdity\left( permeate side \right)}{Thickneess}$	Eq.S2

$J=\frac{d Humidity(permeate side)}{d Time}\times volume of permeate side$	Eq.S3

$J=D\times A\times\frac{d C}{d X}$	Eq.S4

Figure S11. Experiment setup for permeation experiment.

Figure S12. Correlation between concentration difference and vapor flux in PAM-co-PEGMA 8/2 permeation experiment.

Figure S13. Correlation between concentration difference and vapor flux in PAM-co-PEGMA 6/4 permeation experiment.

Figure S14. SEM image of PAM-co-PEGMA 6/4.

# 5. **Thermal Stability of Hydrogel Microspheres**

Figure S15. Thermogravimetric Curve of Hydrogels.

# 6. **Atmospheric Water Harvesting under Controlled Temperature and Humidity Conditions**

Figure S16. Photo of the AWH equipment.

Figure S17. Photo of the environment chamber for the test.

The atmospheric water harvesting equipment, as shown in the figure, mainly consists of a desorption chamber and three adsorption beds. Each bed is loaded with 5×5 cm of MHH membrane.

For the atmospheric water harvesting test, the overall experimental setup is illustrated in the figure. A sealed chamber houses a humidity-controllable humidifier and a temperature-humidity recorder. Externally, a xenon lamp is used to irradiate the desorption chamber, which is located inside the sealed chamber.

To determine the maximum water harvesting capacity of this system, experiments were conducted under conditions of 90% relative humidity (RH) and 25 °C (Figure 6e), with the temperature and humidity variations inside the chamber recorded by the temperature-humidity recorder. Desorption was performed under 1-sun intensity (1000 W m^-2^), where desorption lasted 1 hour, and adsorption lasted 2 hours. The three adsorption beds were alternately moved into the desorption chamber for desorption, thus realizing a semi-continuous atmospheric water harvesting process. The water production was quantified by measuring the mass changes of the adsorption beds and the desorption chamber for each harvesting event.

Figure S18. Outdoor experiment results on August 6, 2026

Figure S19. ICP-OES results of harvest water.

# 7. **Discussion for microbial contamination during water release and collection**

PPy is employed in the MHH membrane primarily as a photothermal coating for solar-driven water desorption. Although antibacterial activity has been reported for PPy-containing materials under certain experimental conditions ^17,18^, no dedicated microbial challenge, colony-counting, or sterility measurements were performed for the present MHH membrane or harvesting device. Therefore, the reported antibacterial behavior of PPy-containing materials should not be considered equivalent to microbiological sterility of the harvested condensate.

Microbial contamination in atmospheric water harvesting systems may additionally arise from air-contact interfaces, condenser surfaces, collection vessels, tubing, and subsequent storage. Recent studies addressing sterile condensate collection have highlighted the importance of controlling the complete condensation, collection, and storage pathway. Practical potable-water production would therefore require appropriate hygienic system design, microbial monitoring, and, where necessary, suitable disinfection or post-collection treatment. The ICP-OES measurements presented in Figure S19 assess inorganic elemental contamination under the tested conditions but do not establish microbiological sterility or complete potability.

# 8. Kinetic Comparison of hygroscopic polymer sorbents from different literatures

To place the sorption kinetics of the present MHH membrane in the context of representative hygroscopic polymer sorbents reported in the literature ^2–11^, the published uptake data were analyzed using a common adsorption kinetic expression where sufficient data were available. The moisture adsorption rate can be evaluated based on the time constant (τ) obtained from curve fitting, where a smaller τ indicates a higher adsorption rate.

$$\frac{W_{t}}{W_{e}}=1-e^{-\frac{t}{\tau}}$$

Table S1 Kinetic Comparison of hygroscopic polymer sorbents

| Hygroscopic polymer sorbents | τ (min) | W_e_ (g/g) | Reference |
| --- | --- | --- | --- |
| MHH membrane | 40.12 | 2.52 | This work |
| Hydrogel microspheres | 110.72 | 2.36 | This work |
| PAM-MXene-LiCl | 207.30 | 4.28 | [2] |
| PAMPS-CNT-LiCI | 94.00 | 1.96 | [3] |
| BPGHM | 61.86 | 2.35 | [4] |
| SA/CNTS/MC | 40.08 | 1.11 | [5] |
| HIPG | 19.46 | 2.00 | [6] |
| LHPE | 187.93 | 3.16 | [7] |
| SBGSs | 39.66 | 1.56 | [8] |
| PCC20@LiCI | 125.61 | 2.85 | [9] |
| CPPY@LiCl | 117.63 | 4.20 | [10] |
| BC/SA/GO-LiCI | 62.96 | 2.39 | [11] |

For contextual benchmarking of device-level water production, representative recently reported SAWH systems with different operating configurations and environmental conditions are summarized in Table S2 ^9,10,12–16^. Because these studies differ substantially in relative humidity, cycle schedule, number of sorbent beds, regeneration conditions, and reporting basis, the comparison is intended to provide context rather than a strict performance ranking

Table S2 Water production comparison in device-level

| Sorbents | Operating mode | Relative Humidity | Water Production | Reference |
| --- | --- | --- | --- | --- |
| MHH membrane | 9 cycles in a day  3 sorbent beds | 63%–73% | 1.964 L/(m^2^ 8hrs) | This work |
| MHH membrane | 9 cycles in a day  3 sorbent beds | 90% | 3.47 L/(m^2^ 8hrs) | This work |
| PVA–LiCl–glycerol hydrogel | 1 cycle in a day  1 sorbent bed | 88% | 1.26 L/(m^2^ d) | [12] |
| CPPY@LiCl | 4 cycles in a day  4 sorbent beds | 60%-70% | 6.29 L/(m^2^ d) | [10] |
| PCC20@LiCl | 9 cycles in a day  4 sorbent beds | 45% | 1.134 L/(m^2^ d) | [9] |
| BHA-213 | 1 cycle in a day  1 sorbent bed | 70.25% | 1.5 L/(m^2^ d) | [13] |
| SBGS | 15 cycles in a day  5 sorbent beds | 60%-80% | 2.38 L/(m^2^ d) | [14] |
| P/LiCl paper | 12 cycles in a day  1 sorbent bed | 65.8% | 0.319 L/(m^2^ d) | [15] |
| PAM@LiCl | 1 cycle in a day  1 sorbent bed | 80%-90% | 2.45 L/(m^2^ d) | [16] |

References

[1] Masaro L, Zhu XX. Physical models of diffusion for polymer solutions, gels and solids. Progress in Polymer Science, 1999, 24: 731-775
[2] Zhou Z, Wang G, Pei X, et al. Solar-driven mxene aerogels with high water vapor harvesting capacity for atmospheric water harvesting. Chemical Engineering Journal, 2023, 474: 145605
[3] Shan H, Poredoš P, Ye Z, et al. All-day multicyclic atmospheric water harvesting enabled by polyelectrolyte hydrogel with hybrid desorption mode. Advanced Materials, 2023, 35: 2302038
[4] Li T, Yan T, Wang P, et al. Scalable and efficient solar-driven atmospheric water harvesting enabled by bidirectionally aligned and hierarchically structured nanocomposites. Nature Water, 2023, 1: 971-981
[5] Zhang X, Qu H, Li X, et al. Autonomous atmospheric water harvesting over a wide RH range enabled by super hygroscopic composite aerogels. Advanced Materials, 2024, 36: 2310219
[6] Yang X, Chen Z, Xiang C, et al. Enhanced continuous atmospheric water harvesting with scalable hygroscopic gel driven by natural sunlight and wind. Nature Communications, 2024, 15: 7678
[7] Zou H, Zhu J, Yang X, et al. High-efficiency atmospheric water harvesting and irrigation recycling in greenhouse using hygroscopic composite gels. Advanced Functional Materials, 2025, 35: 2501163
[8] Zhao Y, Guan W, Wong YZ, et al. Material-to-system tailored multilayer-cyclic strategy toward practical atmospheric water harvesting. Proceedings of the National Academy of Sciences, 2025, 122: e2500928122
[9] Lu J, Yan J, Pei F, et al. Shrinkage-resistant thermo-responsive hygroscopic hydrogel toward ultra-rapid cycling atmospheric water harvesting. Advanced Functional Materials, 2025, 35: 2505359
[10] Lin B, Ying W, Li C, et al. Biomass-derived hydro-sponge for ultra-efficient atmospheric water harvesting. Advanced Functional Materials, 2025, 35: 2500679
[11] Li J, Xu C, Chen L, et al. Tandem atmospheric water harvesting and passive cooling enabled by hygroscopic biopolymer-based aerogels. Advanced Functional Materials, 2025, 35: 2423063
[12] Liu C, Yan X Y, Li S, et al. A metre-scale vertical origami hydrogel panel for atmospheric water harvesting in Death Valley. Nature Water, 2025, 3: 714-722
[13] Zhang L, Yang T, Niu Y, et al. Anisotropic Biomass Microfluidics via Directed Moisture Transport and Enhanced Water‐Binding Capacity for High‐Yield Solar‐Driven Atmospheric Water Harvesting. Advanced Materials, 2026, 38: e22241
[14] Zhao Y, Guan W, Wong Y Z, et al. Material-to-system tailored multilayer-cyclic strategy toward practical atmospheric water harvesting. Proceedings of the National Academy of Sciences, 2025, 122: e2500928122
[15] Guan Y, Tang D, Yu X, et al. Scalable Atmospheric Water Harvesting Paper Enable Rapid and Continuous Water Collection. Advanced Energy Materials, 2026, 16: e70889
[16] Liu Y, Wang J, Li Q, et al. Directional radiative cooling and solar heating-assisted atmospheric water harvesting for all-day water production. Journal of Colloid and Interface Science, 2026: 141171.
[17] Li X, Qiu J, Liu X. Antibacterial property and biocompatibility of polypyrrole films treated by oxygen plasma immersion ion implantation. Advanced Materials Interfaces, 2020, 7: 2000057.
[18] da Silva Jr F A G, Queiroz J C, Macedo E R, et al. Antibacterial behavior of polypyrrole: The influence of morphology and additives incorporation. Materials Science and Engineering: C, 2016, 62: 317-322.
